# Diagnostic Added-Value of Serum CA-125 on the IOTA Simple Rules and Derivation of Practical Combined Prediction Models (IOTA SR X CA-125)

**DOI:** 10.3390/diagnostics11020173

**Published:** 2021-01-26

**Authors:** Phichayut Phinyo, Jayanton Patumanond, Panprapha Saenrungmuaeng, Watcharin Chirdchim, Tanyong Pipanmekaporn, Apichat Tantraworasin, Theera Tongsong, Charuwan Tantipalakorn

**Affiliations:** 1 Department of Family Medicine, Faculty of Medicine, Chiang Mai University, Chiang Mai 50200, Thailand; phichayutphinyo@gmail.com; 2 Center for Clinical Epidemiology and Clinical Statistics, Faculty of Medicine, Chiang Mai University, Chiang Mai 50200, Thailand; jpatumanond@gmail.com; 3 Department of Obstetrics and Gynecology, Faculty of Medicine, Mahasarakham University, Maha Sarakham 44150, Thailand; Pps2pearkabpom@gmail.com; 4 Department of Obstetrics and Gynecology, Phrapokklao Hospital, Chanthaburi 22000, Thailand; watcharin.ch@cpird.in.th; 5 Department of Anesthesiology, Faculty of Medicine, Chiang Mai University, Chiang Mai 50200, Thailand; tanyong24@gmail.com; 6 Department of Surgery, Faculty of Medicine, Chiang Mai University, Chiang Mai 50200, Thailand; ohm_med@hotmail.com; 7 Department of Obstetrics and Gynecology, Faculty of Medicine, Chiang Mai University, Chiang Mai 50200, Thailand

**Keywords:** ovarian cancer, diagnosis, international ovarian tumor analysis (IOTA), ultrasound, CA-125

## Abstract

Background: This study aimed to evaluate the diagnostic added-value of serum CA-125 to the International Ovarian Tumor Analysis (IOTA) Simple Rules in order to facilitate differentiation between malignant and benign ovarian tumors before surgery. Methods: A secondary analysis of a cross-sectional cohort of women scheduled for surgery in Maharaj Nakorn Chiang Mai Hospital between April 2010 and March 2018 was carried out. Demographic and clinical data were prospectively collected. Histopathologic diagnosis was used as the reference standard. Logistic regression was used for development of the model. Evaluation of the diagnostic added-value was based on the increment of the area under the receiver operating characteristic curve (AuROC). Results: One hundred and forty-five women (30.3%) out of a total of 479 with adnexal masses had malignant ovarian tumors. The model that included information from the IOTA Simple Rules and serum CA-125 was significantly more superior to the model that used only information from the IOTA Simple Rules (AuROC 0.95 vs. 0.89, *p* < 0.001 for pre-menopause and AuROC 0.98 vs 0.83, *p* < 0.001 for post-menopause). Conclusions: The IOTA SR X CA-125 model showed high discriminative ability and is potentially useful as a decision tool for guiding patient referrals to oncologic specialists.

## 1. Introduction

General gynecologists are required to provide an accurate differentiation between benign and malignant adnexal pathologies to ensure an optimal starting point in the whole chain of care [1], as this would lead to appropriate decisions regarding the referral of patients to specialized oncologic care. Women with malignant masses should be referred to gynecologic oncologists for proper surgical staging and optimal debulking surgery [2]. In contrast, women with benign masses can be managed conservatively or with a minimally invasive approach (e.g., laparoscopic surgery), which can be safely performed by general gynecologists. Misclassification in either direction could ultimately lead to a decrease in patient survival, or serious morbidity and unnecessary infertility from overly radical surgery.

Transvaginal ultrasonography is generally the first modality used by gynecologists to characterize these masses in practice. It is currently the only imaging modality recommended by the American College of Obstetricians and Gynecologists (ACOG) to evaluate adnexal masses in women [3]. To date, this is widely accepted that the most accurate approach for the preoperative diagnosis of adnexal masses is subjective assessment (SA) by an experienced sonographer [4]. It has been proved superior to other widely advocated methods such as the Risk of Malignancy Index (RMI), the International Ovarian Tumor Analysis (IOTA) Simple Rules, and IOTA logistic regression models [4,5]. However, there are still major limitations with the subjectivity of such a method and the lack of expert examiners in most settings.

The IOTA models carry important advantages over subjective assessments in terms of objectivity, simplicity, and applicability [6]. They provide easy-to-use guidance to non-expert sonographers for making an accurate presurgical diagnosis. Many multi-national external validation studies have confirmed the robustness of the accuracy of the IOTA Simple Rules and the IOTA logistic regression models [7,8]. The main disadvantage of the IOTA Simple Rules is the possibility of inconclusive results when they do not apply. According to previous reports, the proportion of inconclusive results could be as high as 20% [9,10]. A “two-step strategy” using the IOTA Simple Rules with the addition of subjective assessment for masses with inconclusive results was proposed and proved to have excellent test performance comparable to that of subjective assessment alone [11]. However, this strategy requires the availability of experienced examiners in the same setting to avoid unnecessary referrals and to reduce the health care costs.

Serum cancer antigen 125 or CA-125 is a well-known biomarker for epithelial ovarian cancer. Although the role of CA-125 in the diagnosis of ovarian cancer is controversial due to its only fair level of sensitivity and poor specificity, it is still widely used in the assessment of women with adnexal masses and is routinely used in preoperative investigation. Other biomarkers have been investigated to improve specificity for the diagnosis of ovarian cancer, such as the Human Epididymis Protein 4 or HE4. Although HE4 is highly specific to ovarian malignancy, it is not as sensitive as serum CA-125 [12]. For this reason, the risk of malignancy algorithm (ROMA) was developed to incorporate both the sensitivity of serum CA-125 and the specificity of HE4 to yield a better diagnostic performance [13]. However, in Thailand, HE4 analysis is not generally used in the preoperative evaluation due to its relatively high cost and the fact that it is only available in few institutions.

For pre-surgical diagnosis of women with adnexal masses, the American College of Obstetrics and Gynecology recommends a multivariable approach by combining demographic, clinical, laboratory, and imaging parameters to achieve better diagnostic accuracy [3,14]. Even though the application of IOTA Simple Rules has recently been proven acceptably accurate in our setting [9], we hypothesized that its accuracy could be further improved by including other relevant parameters such as serum CA-125. The primary aim of this study was to evaluate the diagnostic added-value of serum CA-125 to the IOTA Simple Rules, without subjective assessment, to differentiate between malignant and benign ovarian tumors before surgery. The evaluation would be done separately for premenopausal and postmenopausal women to eliminate the presence of effect modification. The secondary aim was to derive new prediction models based on the IOTA Simple Rules and serum CA-125 to enable the diagnostic prediction of ovarian malignancy in all patients, both conclusive and inconclusive, without relying on the presence of experienced sonographers.

## 2. Materials and Methods

### 2.1. Design and Setting

A secondary analysis of a cross-sectional cohort of women with adnexal masses was performed to evaluate the diagnostic added-value of serum CA-125 and to develop novel diagnostic models for the prediction of ovarian malignancy. The data of patients who were admitted for pelvic operation for adnexal masses at Maharaj Nakorn Chiang Mai Hospital were prospectively collected between April 2010 and March 2018. The hospital is a university-affiliated teaching hospital with a specialized oncologic center located in Chiang Mai Province.

### 2.2. Study Patients and Data Collection

Women with adnexal masses who were scheduled for surgery that met the following criteria were included in the analysis: (1) were diagnosed with an adnexal mass by either pelvic ultrasonographic examination or by vaginal examination; (2) had no known diagnosis of the mass before surgery. For women who had more than one adnexal mass, the mass with most complex ultrasonographic features, or the mass with higher malignancy potential on sonographers’ judgment was included. Patients whose mass was surgically removed after 24 h of ultrasonographic examination and patients without preoperative CA-125 were excluded. Preoperative transvaginal ultrasound examination was performed in all included patients by non-expert sonographers. Sonographers were blinded to patients’ clinical characteristics and preoperative laboratory results. During ultrasonographic examination, the morphology of the adnexal masses was characterized using 2D real-time and color Doppler ultrasound.

Demographic and clinical data including age, parity, menopausal status, and tumor marker level (i.e., CA-125) were prospectively collected. Ultrasonographic features of the adnexal masses based on the IOTA Simple Rules and the ultrasound score of the Risk of Malignancy Index (RMI) were recorded.

### 2.3. The IOTA Simple Rules

The International Ovarian Tumor Analysis (IOTA) Simple Rules is a classification system for preoperative diagnosis of ovarian cancer [15]. According to these rules, the adnexal masses are categorized into benign, malignant, or inconclusive tumors based on the presence of benign features (B-features) or malignant features (M-features). The B-features are as follows: (1) unilocular; (2) presence of solid components with largest diameter <7 mm; (3) presence of acoustic shadows; (4) smooth multilocular tumor with largest diameter <100 mm; and (5) no blood flow (color score 1). The M-features are as follows: (1) irregular solid tumor; (2) presence of ascites; (3) at least four papillary structures; (4) irregular multilocular-solid tumor with largest diameter ≥100 mm; and (5) very strong blood flow (color score 4). The mass would be categorized as benign if one or more B-features applied in the absence of an M-feature. Conversely, the mass would be categorized as malignant if one or more M-features applied in the absence of a B-feature. If both M-features and B-features applied or neither of the features applied, the mass was categorized as inconclusive.

### 2.4. Reference Standard

Histopathologic diagnosis of the surgical specimen was used as the reference standard for definite diagnosis of the adnexal masses. In the case of some benign masses without pathological specimens, intraoperative diagnosis made by the surgeons was used as reference. All adnexal masses were classified into two groups, benign or malignant ovarian tumors. Borderline ovarian tumors were grouped with malignant ovarian tumors.

### 2.5. Statistical Analysis

Statistical analyses were carried out using Stata version 16 (StataCorp, College Station, TX, USA). Frequency and percentage were used to describe categorical data. Mean and standard deviation or median and interquartile range were used to express continuous data according to their distribution. An exact probability test was used to compare the differences in categorical data between groups. An independent *t*-test or a Mann-Whitney U test was used to compare the differences in continuous data as appropriate.

#### 2.5.1. Evaluation of Diagnostic Added-Value

The following steps were performed to evaluate the diagnostic added-value of CA-125 to the IOTA Simple Rules in premenopausal and postmenopausal women. First, three logistic regression models were developed separately in each subgroup of women. The results of the IOTA Simple Rules were left in their three original categories, without exclusion of patients with inconclusive results. This allowed us to utilize the full data of the patients. The first model included only the information from the IOTA Simple Rules (PRE1 and POST1). The second model included only the information from the log-transformed CA-125 (PRE2 and POST2). The third model included the information from both the IOTA Simple Rules and the log-transformed CA-125 (PRE3 and POST3). For both the second and the third model, a multivariable fractional polynomials (MFP) algorithm was performed to best fit the continuous value of the log-transformed CA-125 into the binary logistic model. In this study, the log-transformed CA-125 was included as the first-degree fractional polynomial term (FP1), log (CA-125)^3^.

The area under the receiver operating characteristic curve (AuROC) was used as the main measure of model performance. We employed the method proposed by DeLong and colleagues to check for significant differences in AuROCs [16]. If the AuROCs of the PRE3 and POST3 models were significantly better than the AuROC of the PRE1 and POST1 models, we concluded that serum CA-125 had added diagnostic value to the IOTA Simple Rules. In contrast, if the AuROCs of the PRE3 and POST3 models were not significantly better than the AuROC of the PRE1 and POST1, we concluded that serum CA-125 did not add any diagnostic value to the IOTA Simple Rules.

#### 2.5.2. Prediction Model Development

If the models that included both serum CA-125 and the IOTA Simple Rules outperformed the models that contained only the IOTA Simple Rules, the combined models were developed further into a diagnostic prediction model and evaluated for model diagnostic performance. We plan to develop separate models for premenopausal and postmenopausal women in order to eliminate the modifying effect of menopausal status. The models were then presented as logistic regression equations. The prediction of probability of ovarian malignancy can be estimated by the inverse logit transformation of the linear predictor (lp) as follows: probability = e^lp^/(1 + e^lp^), where e is the base value of natural logarithms.

#### 2.5.3. Prediction Model Performance

The performance of each prediction model was measured separately in terms of discrimination, calibration, and clinical utility. The measure of discrimination was AuROC as described earlier. To examine the agreement between the model predicting the risk of malignancy and the observed proportion of malignancy, calibration plots were completed. Internal validation was performed using a boot-strap procedure with 500 replicates. The model optimism and shrinkage factor were estimated and presented

To evaluate the clinical utility of the combined IOTA Simple Rules and CA-125 models over the models using IOTA Simple Rules alone, we conducted a decision curve analysis (DCA) [17]. This simple approach focuses on the net benefit (NB) gained from using the prediction models in making clinical decisions. The NB is calculated as the subtraction of harms (false positives) from benefits (true positives), as in the subtraction of expenditure from total income to calculate profit [18]. The decision curves were plotted to visualize the trend in NB of the prediction models across the range of threshold probability of patient referrals. The threshold probability is the minimum probability of ovarian malignancy at which a general gynecologist would opt for referral to oncologists. The NB of the index models should be compared to the two default strategies of referring all patients or not referring any patients. A prediction model with a clinical usefulness should have higher value of NB over the other models and the two default strategies across the entire range of threshold probability. In the context of pre-surgical diagnosis of ovarian cancer, we chose 5% to 50% as reasonable threshold probabilities. These decisions were made using the simple guidance on the interpretation of DCA in a document which was recently published by the pioneers of the methods [19].

#### 2.5.4. Diagnostic Accuracy of the Models

Sensitivity, specificity, positive predictive values (PPV), negative predictive values (NPV), and positive likelihood ratios (LHR+) were calculated to compare the diagnostic ability of the IOTA Simple Rules and the newly derived prediction models at different levels of risk threshold. For binary classification of the IOTA Simple Rules, masses that were interpreted as malignant and inconclusive were classified as malignancy. In the case of the combined prediction models, we pre-specified the risk-thresholds for evaluation of diagnostic ability at ≥10%, ≥20%, ≥30%, ≥40%, and ≥50%. The analyses were done separately using the premenopausal and postmenopausal data.

## 3. Results

### 3.1. Baseline Characteristics of the Study Patients

Out of a total of 479 women with adnexal masses included in this secondary analysis, 145 (30.3%) had malignant ovarian tumors and 334 (69.7%) had benign ovarian tumors. There were 115 (24.0%) postmenopausal women and 364 (76.0%) premenopausal women. The comparison of clinical characteristics, biomarkers, and features of the IOTA Simple Rules between women with malignant and benign ovarian tumors are presented in Table 1. There were significant differences in the proportions of nulliparous women (51.7% vs. 41.0%, *p* = 0.035), and postmenopausal women (40.0% vs. 17.1%, *p* < 0.001) between groups. All aspects of the IOTA Simple Rules (the M features and the B-features) showed a significant difference between women with malignant and benign ovarian tumors. However, in this cohort, the IOTA Simple Rules can only be applied in 392 (81.8%) women with conclusive results. The proportion of inconclusive results was not significantly different between malignant and benign tumors (19.3% vs. 17.7%, *p* = 0.699)

The differences in clinical characteristics between women with malignant and benign ovarian tumors for premenopausal and postmenopausal women are presented in Supplementary Materials Tables S1 and S2, respectively. Supplementary Materials Table S3 shows histopathological classification of ovarian tumors in both premenopausal and postmenopausal women.

### 3.2. The Added-Value of CA-125

**Figure 1:**
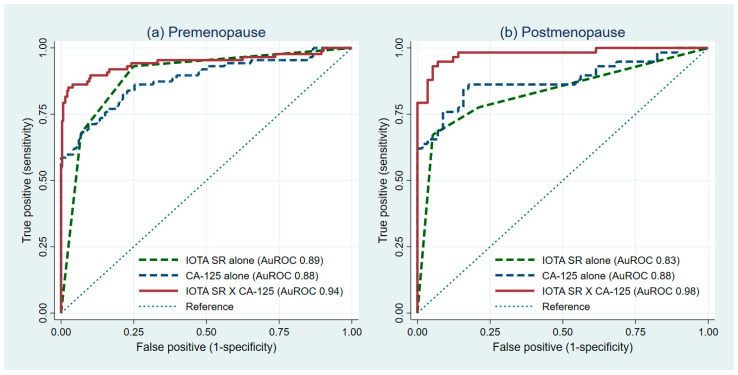
Receiver operating characteristic curves from the model with IOTA SR alone, CA-125 alone, and combined IOTA SR and CA-125 models (IOTA SR X CA-125) for premenopausal and postmenopausal women. IOTA SR, International Ovarian Tumor Analysis Simple Rules. Evaluation of diagnostic added-value of CA-125 in (**a**) premenopausal women and (**b**) postmenopausal women.

The evaluation of the impact of the diagnostic value of serum CA-125 on the IOTA Simple Rules for premenopausal and postmenopausal women is shown in Table 2. The ability of the PRE1, PRE2, and PRE3 model to discriminate between malignant ovarian tumors and benign ovarian tumors via AuROC were 0.89 (95%CI 0.86, 0.93), 0.88 (95%CI 0.83, 0.93), and 0.94 (95%CI 0.91, 0.98), respectively (Figure 1a). The ability of the POST1, POST2, and POST3 model via AuROC were 0.83 (95%CI 0.76, 0.90), 0.88 (95%CI 0.81, 0.95), and 0.98 (95%CI 0.95, 1.00), respectively (Figure 1b). The results on the pairwise comparison between the first (PRE1 and POST1) and the third model (PRE3 and POST3) are presented in Table 3. In both premenopausal and postmenopausal women, the model that uses the information from the IOTA Simple Rules and CA-125 (PRE3 and POST3) was significantly superior than the model that uses only the information from the IOTA Simple Rules (PRE1 and POST1) (Table 3).

### 3.3. Prediction Model Performance

In the case of premenopausal women, the PRE3 model exhibited the best discriminative ability (AuROC 0.94, 95%CI 0.91, 0.98). It contained only two sets of predictors: (1) the result of IOTA Simple Rules; and (2) the log-transformed CA-125. In premenopausal women, the probability of ovarian malignancy can be estimated from the PRE3 model via the following equation: e^lp^/(1 + e^lp^), where lp = −4.011 + 0 (benign result from IOTA Simple Rules) + 2.101 (inconclusive result from IOTA Simple Rules) + 4.266 (malignant result from IOTA Simple Rules) + 0.023 (log-transformed CA-125) (Table 2).

In postmenopausal women, the POST3 model exhibited the best discriminative ability (AuROC 0.98, 95%CI 0.95, 1.00). It contained only two predictors: (1) the result of IOTA Simple Rules; and (2) the log-transformed CA-125. In postmenopausal women, the probability of ovarian malignancy can be estimated from the POST3 model via the following equation: e^lp^/(1 + e^lp^) where lp = −2.225 + 0 (benign result from IOTA Simple Rules) + 0.7127 (inconclusive result from IOTA Simple Rules) + 5.296 (malignant result from IOTA Simple Rules) + 0.030 (log-transformed CA-125) (Table 2).

**Figure 2:**
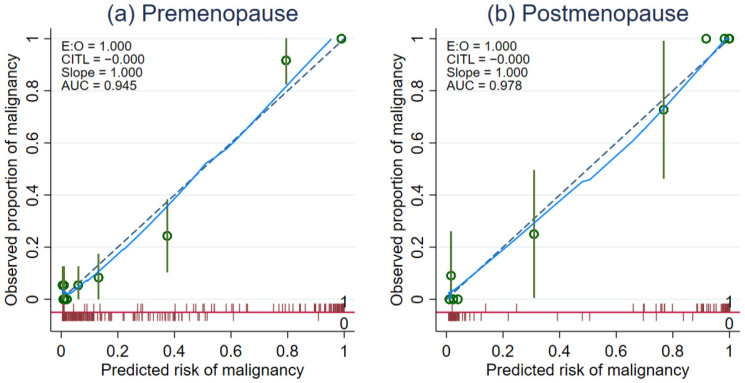
Calibration plots examining agreement between predicted risk of malignancy from the combined IOTA Simple Rues models (IOTA SR X CA-125) for premenopausal and postmenopausal women and the observed proportion of malignancy stratified by menopausal status. Evaluation of model calibration in (**a**) premenopausal women and (**b**) postmenopausal women. The diagonal green dash line is given as a reference for perfect model calibration. The observed proportion of ovarian malignancy at each coordinating predicted risk level is shown with green hollow circles with confidence intervals. The blue line is a lowess smoother visualizing the overall trend of calibration. The spike plot is shown in red to visualize the distribution of events and non-events across the predicted risk of malignancy.

For the premenopausal model, the apparent AuROC was 0.9481 and the test AuROC was 0.9442. The optimism was estimated at 0.0039 and the shrinkage factor was 0.9821. In the postmenopausal model, the apparent AuROC was 0.9771 and the test AuROC was 0.9761. The optimism was estimated at 0.0010 and the shrinkage factor was 0.9751. The detailed results of the internal bootstrap validation procedure are shown in Supplementary Materials Table S4. The agreement between the predicted probability of ovarian malignancy from both prediction models and the observed proportion of malignancy in each group of women was visualized from the calibration plot (Figure 2a,b).

**Figure 3:**
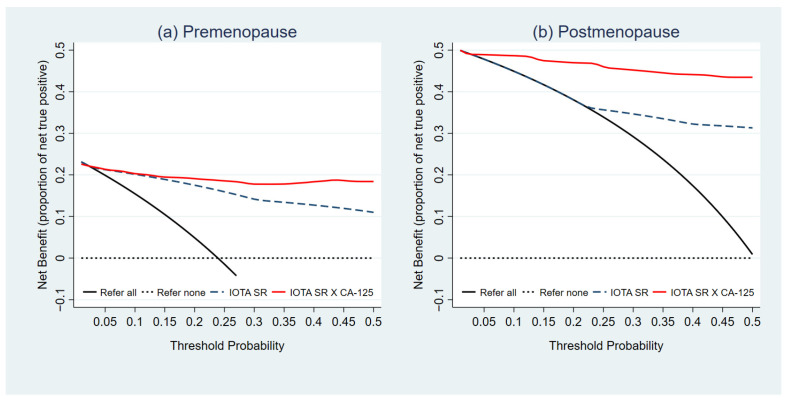
Decision curves representing the net benefit (NB) of the combined IOTA SR models for premenopausal and postmenopausal women. Evaluation of clinical usefulness in (**a**) premenopausal women and (**b**) postmenopausal women.

The clinical utility of the prediction models was illustrated via the decision curve (Figure 3a,b). The NB of both the IOTA Simple Rules and PRE3 model, or the IOTA SR X CA-125 in premenopausal women, was higher than the default strategies, specifically an approach to refer all patients or not to refer any patient, across the entire range of threshold probability for patient referrals. The NB of the combined model was higher than that of the IOTA Simple Rules beyond the threshold probability of 15%. The NB of the POST3 model or the IOTA SR X CA-125 model for postmenopausal women was higher than both default strategies and the IOTA Simple Rules alone across the entire range of threshold probability for patient referrals. The NB of the IOTA Simple Rules started to depart from the approach to refer all patients after a threshold probability of 20%.

### 3.4. Comparative Validation of Diagnostic Performance

In premenopausal women, only 80.2% (292/364) had conclusive IOTA Simple Rules results. The sensitivity and specificity of the IOTA Simple Rules in women with conclusive results were 90.8% (95%CI 81.0%, 96.5%) and 91.6% (95%CI 87.2%, 94.9%), respectively. By considering referral of patients with an inconclusive result, the sensitivity of the IOTA Simple Rules increased to 93.1% (95%CI 85.6%, 97.4%), whereas the specificity dropped to 75.1% (95%CI 69.6, 80.1%) (Table 4). The sensitivity and specificity of the IOTA SR X CA-125 model for premenopausal women differ depending on the selected risk threshold to predict malignancy. The sensitivity, specificity, positive predictive values, negative predictive values, and positive likelihood ratios at each pre-specified risk threshold of the IOTA SR X CA-125 model are presented in Table 4.

In postmenopausal women, only 87.0% (100/115) had conclusive IOTA Simple Rules results. The sensitivity and specificity of the IOTA Simple Rules in women with conclusive results were 75.0% (95%CI 61.1%, 86.0%) and 93.8% (95%CI 82.8%, 98.7%), respectively. When women with inconclusive results were considered for referral, as in the case of women with malignant results, the sensitivity of the IOTA Simple rules increased to 77.6% (95%CI 64.7%, 87.5%), whereas the specificity dropped to 78.9% (95%CI 66.1%, 88.6%) (Table 5). The sensitivity and specificity of the IOTA SR X CA-125 model for postmenopausal women differ depending on the selected risk threshold to predict malignancy. The sensitivity, specificity, positive predictive values, negative predictive values, and positive likelihood ratios at each pre-specified risk threshold of the IOTA SR X CA-125 model are presented in Table 5.

## 4. Discussion

In this study, the addition of serum CA-125 to the IOTA Simple Rules was proven to increase the diagnostic value in preoperatively differentiating between malignant and benign ovarian tumors in women who presented with adnexal masses. The benefit of such an approach was identified in both premenopausal and postmenopausal women. However, the improvement in diagnostic performance seems to be larger in postmenopausal women. The prediction models that combine the information from both the IOTA Simple Rules and serum CA-125, or the IOTA Simple Rules X CA-125 models, might be comparable to the widely accepted two-step strategy of IOTA Simple Rules and the IOTA logistic regression models. The application of the combined models in practice might be a more practical and effective approach for the triage of women with adnexal masses in settings where experienced sonographers were not available to provide accurate subjective evaluations of the masses.

Within the past decade, the IOTA Simple Rules have gained more popularity and have been continuously validated and implemented in many academic hospitals [20], including our institution [21,22]. According to one meta-analysis, the pooled sensitivity and specificity of the IOTA Simple Rules was 93.0% and 95.0%, respectively [23]. However, the rate of inconclusive results from the IOTA Simple Rules was relatively high, 10%–20% on average [9,23,24]. In the case of non-academic hospitals, where experienced sonographers were not generally available, the patients with inconclusive results still needed to be referred to specialized oncologic centers (i.e., to be managed in the same way as patients with malignant results from the rules). In our previous validation study on the IOTA Simple Rules [9], the sensitivity and specificity of the rules where only patients with conclusive results were included were 83.8% and 92.0%, respectively. In this study, to which the same dataset was applied, when inconclusive patients were interpreted as malignant patients, the accuracy substantially changed. In postmenopausal women, the sensitivity and specificity of the IOTA Simple Rules dropped to 77.6% and 78.9%, respectively. In contrast, the sensitivity increased to 93.1% and the specificity decreased to 75.1% in premenopausal women.

According to a recent systematic review and meta-analysis of 47 studies, it is suggested that a two-step strategy should be used for patients with inconclusive results from the IOTA Simple Rules to achieve the highest level of diagnostic accuracy (sensitivity 91.0% and specificity 91.0%) [11]. In circumstances where an expert is not available, the IOTA logistic regression model 2 (LR2) can be used as an alternative to the IOTA Simple rules with subjective assessment, as either approach would ultimately result in comparable diagnostic performance [1,11]. However, compared to the IOTA Simple Rules, the IOTA logistic models required more detailed information for each predictor and were not as easy to memorize and execute. We proposed an alternative approach to the use of the IOTA logistic models by using complete information from the IOTA Simple Rules results together with serum CA-125 level to predict the probability of ovarian malignancy. With this approach, the risk can be accurately predicted from the combined models in all patients regardless of their IOTA Simple Rules results.

The role of serum CA-125 in ovarian cancer diagnosis is subject to controversy [1]. Despite its limitations, serum CA-125 is still the most widely used marker for epithelial ovarian cancer. Studies had reported variation in the discriminative performance of serum CA-125. One study in Oman reported an AuROC of serum CA-125 at 0.75 for ovarian cancer diagnosis [25]. Another study in Turkey, reported an AuROC of 0.78 [26]. A recent meta-analysis of 19 studies which examined the diagnostic performance of serum CA-125 in Chinese patients reported a high AuROC of 0.84 [27], which was considered acceptable. However, most studies examined the diagnostic accuracy and discriminative performance of serum CA-125 at specific cut-off values, most commonly at the standard established cutoff at ≥35 U/mL [28]. Dichotomization of continuous variables results in significant losses of information and may lead to a spurious predictor-outcome relationship, which could substantially affect the discriminative performance of the biomarkers [29,30]. In this study, we avoided dichotomizing the CA-125 values by employing a fractional polynomials procedure for flexible modeling of potential non-linear association between serum CA-125 and probability of ovarian malignancy [31].

In this study, fractional-polynomials transformed values of serum CA-125 alone had excellent discriminative ability in both premenopausal and postmenopausal women (AuROC 0.88 for both groups). This was not in accord with previous studies on the diagnostic value of CA-125 [1,24,32], which were claimed to be higher in postmenopausal women and lower in premenopausal women. The discordance in discriminative ability of serum CA-125 in our study may be explained by the clinical heterogeneity of patients recruited in each study and a different mix of ovarian tumors in premenopausal and postmenopausal women [32,33]. In a recent study in Italy which examined the influences of biomarkers on the diagnostic performance of the IOTA Simple Rules [24], the proportion of malignant mass was 10% in premenopausal women and 37% in postmenopausal women, whereas the proportion of malignant mass was 20.8% in premenopausal and 46.9% in post-menopausal in our study. In addition, the pattern of serum CA-125 in benign and malignant tumors was similar in premenopausal and postmenopausal women.

One study by a team investigating models and ovarian cancer examined the added-value of serum CA-125 to the mathematical prediction models in differentiating between benign and malignant adnexal tumors and concluded that the measurement of serum CA-125 was unnecessary, especially in premenopausal women [32]. The study reported an average AuROC of serum CA-125 at 0.81. The stratified analysis revealed that the AuROC was 0.63 in premenopausal women and 0.92 in postmenopausal women. The higher proportions of patients with borderline tumors in that study in comparison to our study (21.5% vs. 10.3%) might partially explain the difference in CA-125 performance. Other tumor characteristics may also affect the discriminative ability of serum CA-125, such as the histologic grades, the presence of extraovarian invasions, and the proportion of patients with early-stage ovarian cancer [34]. Another important point raised by the authors was that other ultrasound predictors (e.g., presence of ascites, maximum tumor diameter, and maximum diameter of solid component) included within the model were significantly more informative than serum CA-125 in characterizing adnexal tumors [32].

A more recent study in Italy examined the added-value of serum CA-125 to the IOTA Simple Rules two-step approach and concluded that the addition of serum CA-125 to such strategies increased the net reclassification index and was cost-effective among postmenopausal women [24]. In our study, the diagnostic value of the combination of serum CA-125 with full information from the IOTA Simple Rules seemed to be more significant in postmenopausal women than in premenopausal women, as the IOTA Simple Rules was more effective in premenopausal women than postmenopausal women (AuROC 0.89 vs. 0.83). This agreed with the subgroup analyses of a recent meta-analysis in 2014, which showed a higher accuracy of the IOTA Simple Rules in premenopausal women [8]. In our study, the superiority of the IOTA Simple Rules in premenopausal women was obviously the result of a higher proportion of endometriotic cysts, mature cystic teratomas, and pseudocysts in premenopausal women. These benign pathologies were shown to be diagnosed more correctly by the IOTA Simple Rules, either with or without subjective assessment [24].

There were four primary strengths to our combined IOTA Simple Rules and serum CA-125 prediction models. First, the models were derived separately from data from premenopausal and postmenopausal women. As the effect modification of menopausal status can substantially affect the diagnostic model performance, development of a prediction model for each specific group of women may result in a more accurate prediction. Second, these models effectively utilize full information from the IOTA Simple Rules by considering and incorporating inconclusive results, as one diagnosis category, into the models. Third, the newly developed models combined the advantages of both ultrasound and biomarker approaches in the prediction of ovarian risk and were equipped with a high diagnostic accuracy comparable to the IOTA LR2 with significantly fewer numbers of predictors (AuROC 0.94 vs. 0.94 in premenopausal and AuROC 0.98 vs. 0.93 in postmenopausal women) [7]. Considering only the two-step strategy, our models had higher discriminative ability (AuROC 0.94 vs. 0.90 in premenopausal and AuROC 0.98 vs. 0.80 in postmenopausal women) [24]. Lastly, based on the decision curve analysis, the combined model with serum CA-125 was also proven to be clinically more useful than use of the model with IOTA Simple Rules alone in both premenopausal and postmenopausal women.

There are some limitations to be addressed. First, the data on the clinical staging of ovarian cancer was not available, as the study was originally intended to evaluate only the accuracy of IOTA ultrasound parameters for ovarian cancer diagnosis. Thus, we could not fully explain the discrepancy between our results and those in other studies in terms of staging. Second, a head-to-head comparative validation of the combined models with IOTA logistic models or a two-step approach cannot be performed in our dataset as some of the essential predictors were not collected. Third, in our study, ultrasound examiners were obstetrics and gynecologic residents in training with varying levels of sonographic experience. For this reason, their IOTA Simple Rules interpretation might have the same level of accuracy as that of experienced sonographers. However, we believe that the results could be viewed as pragmatic and could more closely resemble the situation in the real world as regards clinical examiners. Fourth, this was a secondary analysis of patient database which was not intended to be stratified by menopausal status. Therefore, the study size might not be adequate and might result in model overfitting. However, based on our post hoc estimation, the number of outcome events per variable (EPV) exceeded 10 for both premenopausal and postmenopausal groups [30,35]. Finally, the derivation of both models was based on a dataset from a single oncologic center that might not be a representative sample, generalizable across an entire population. A larger, multi-center external validation study is warranted before the models be considered for clinical implementation.

For implementation, clinical applicability of our diagnostic models is mathematically straightforward. The logistic regression models require data from only two main predictors, the results of the IOTA Simple Rules and serum CA-125, to estimate the probability of ovarian malignancy (Table 2). There are three possible interpretations for the IOTA Simple Rules: benign, inconclusive, and malignant. Each interpretation is assigned with a specific log odds ratio for calculation. Serum CA-125 will have to be log-transformed, cubed, and re-centered before being put into the equation. Given a case of a premenopausal woman with an IOTA Simple Rules inconclusive result and a serum CA-125 level of 325 U/mL, one can calculate the probability by, first, calculating the linear predictors from the premenopausal model (linear predictors = −4.011 + 2.101 (inconclusive results) + 0.023 (log (325)^3^-72.7142)). Then, use the inverse logit function to convert linear predictors into the probability scale. The calculated probability from the premenopausal model was 70.4%. At a predicted probability ≥50%, the likelihood ratio of her mass being malignant is extremely high (LR + 56.51). Therefore, she should be referred to a gynecologic oncologist for proper evaluation. In practice, our logistic models could be further developed into a user-friendly application for ease of use.

## 5. Conclusions

In conclusion, this study demonstrated that serum CA-125 significantly adds value to the IOTA Simple Rules in differentiating malignant adnexal masses from benign. We also developed the diagnostic models by incorporating serum CA-125 levels into the well-accepted IOTA Simple Rules, entitled IOTA SR X CA-125. We presented the models as logistic regression equations, which estimate the probability of malignancy for each mass. Several probability cutoff points were proposed to guide gynecologists in patient referrals for clinical applicability. Our simple models are able to provide accurate presurgical diagnosis and are potentially useful in reducing inappropriate referrals. We particularly support the implementation of our models in practice, especially in settings where there are no specialized oncologists or experienced sonographers to make final interpretations in cases of inconclusive results from the IOTA Simple Rules.

## Figures and Tables

**Table 1:** Patient clinical characteristics and IOTA Simple Rules results (*n* = 479).

Parameters	Malignant (*n* = 145)		Benign (*n* = 334)		*p*-Value
	*n*	(%)	*n*	(%)	
Clinical characteristics					
Age (year) *	45.4	±14.8	40.6	±11.0	<0.001
Nulliparity	75	(51.7)	137	(41.0)	0.035
Post-menopause	58	(40.0)	57	(17.1)	<0.001
Biomarker					
Serum CA-125, (U/mL) **	405.8	(122.4, 714.8)	39.0	(23.6, 56.9)	<0.001
Range (min-max)		(13.7–2023.6)		(5.4–278.3)	
IOTA Simple Rules features					
Malignant tumor (M-features)					
M1	73	(50.3)	16	(4.8)	<0.001
M2	34	(23.5)	11	(3.3)	<0.001
M3	32	(22.1)	26	(7.8)	<0.001
M4	74	(51.0)	26	(7.8)	<0.001
M5	96	(66.2)	35	(10.5)	<0.001
Benign tumor (B-features)					
B1	9	(6.2)	169	(50.6)	<0.001
B2	1	(0.7)	17	(5.1)	0.018
B3	5	(3.5)	66	(19.8)	<0.001
B4	17	(11.7)	78	(23.4)	0.004
B5	42	(29.0)	297	(88.9)	<0.001
IOTA Simple Rules diagnosis					
Benign	19	(13.1)	253	(75.8)	NA
Malignant	98	(67.6)	22	(6.6)	NA
Inconclusive	28	(19.3)	59	(17.6)	0.699

Abbreviations: IOTA, International Ovarian Tumor Analysis; SD, standard deviation; IQR, interquartile range; NA, not applicable. * Mean ± SD ** Median (IQR).

**Table 2:** Evaluation of the diagnostic value of serum CA-125 to the IOTA Simple Rules for premenopausal and postmenopausal women.

Models	Predictors Included	ß	(95%CI)	*p*-Value	AuROC (95%CI)
Premenopausal women (*n* = 364)					
PRE1	IOTA Simple Rules				
	Benign	Ref	Ref		0.89 (0.86, 0.93)
	Inconclusive	2.72	(1.77, 3.68)	<0.001	
	Malignant	4.68	(3.72, 5.64)	<0.001	
PRE2	Log serum CA-125 (FP1) §	0.02	(0.01, 0.03)	<0.001	0.88 (0.83, 0.93)
PRE3	IOTA Simple Rules				
	Benign	Ref	Ref		0.94 (0.91, 0.98)
	Inconclusive	2.10	(0.99, 3.21)	<0.001	
	Malignant	4.27	(3.11, 5.42)	<0.001	
	Log serum CA-125 (FP1) §	0.02	(0.01, 0.03)	<0.001	
	Intercept (constant)	−4.01	(−4.95,−3.06)	<0.001	
Postmenopausal women (*n* = 115)					
POST1	IOTA Simple Rules				
	Benign	Ref	Ref		0.83 (0.76, 0.90)
	Inconclusive	0.84	(−0.37, 2.04)	0.173	
	Malignant	3.81	(2.48, 5.13)	<0.001	
POST2	Log serum CA-125 (FP1) †	0.02	(0.01, 0.03)	<0.001	0.88 (0.81, 0.95)
POST3	IOTA Simple Rules				
	Benign	Ref	Ref		0.98 (0.95, 1.00)
	Inconclusive	0.71	(−1.63, 3.05)	0.551	
	Malignant	5.30	(3.17, 7.43)	<0.001	
	Log serum CA-125 (FP1) †	0.03	(0.01, 0.04)	<0.001	
	Intercept (constant)	−2.23	(−3.52,−0.93)		

Abbreviations: ß, beta-coefficient of logistic regression; IOTA, International Ovarian Tumor Analysis; Ref, reference; FP1, first-degree fractional polynomial; CI, confidence interval; AuROC, area under the receiver operating characteristic curve. § a re-centered FP1 term for premenopausal model: log (CA-125)^3^−72.7142 † a re-centered FP1 term for postmenopausal model: log (CA-125)^3^−97.7399.

**Table 3:** Diagnostic added-value of serum CA-125 over the IOTA Simple Rules in premenopausal and postmenopausal women.

Comparison	AuROC	*p*-Value *	Log Likelihood	*p*-Value **
Premenopausal women (*n* = 364)				
PRE1 vs. PRE3	0.89 vs. 0.95	<0.001	−114.9805 vs.−74.2167	<0.001
Postmenopausal women (*n* = 115)				
POST1 vs. POST3	0.83 vs. 0.98	<0.001	−51.76 vs.−21.1981	<0.001

Abbreviations: AuROC, area under the receiver operating characteristic curve. * *p*-value for significant difference in AuROC using method proposed by DeLong et al. ** *p*-value from likelihood-ratio test.

**Table 4:** Diagnostic indices of the combined IOTA Simple Rules models for premenopausal women.

	Malignant	Benign	Sensitivity (%)	Specificity (%)	PPV (%)	NPV (%)	LR+
	*n*	*n*	(95%CI)	(95%CI)	(95%CI)	(95%CI)	(95%CI)
Premenopausal women (*n* = 364)							
IOTA SR–refer women with inconclusive results							
Malignant and inconclusive	81	69	93.1	75.1	54.0	97.2	3.74
Benign	6	208	(85.6, 97.4)	(69.6, 80.1)	(45.7, 62.2)	(94.0, 99.0)	(3.02, 4.62)
IOTA SR X CA-125 for premenopausal women							
Predicted risk ≥10%	80	59	92.0	78.7	57.6	96.9	4.32
	7	218	(84.1, 96.7)	(73.4, 83.4)	(48.9, 65.9)	(93.7, 98.7)	(3.41, 5.46)
Predicted risk ≥20%	78	34	89.7	87.7	69.6	96.4	7.30
	9	243	(81.3, 95.2)	(83.3, 91.3)	(60.2, 78.0)	(93.3, 98.4)	(5.29, 10.09)
Predicted risk ≥30%	75	25	86.2	91.0	75.0	95.5	9.55
	12	252	(77.1, 92.7)	(87.0, 94.1)	(65.3, 83.1)	(92.2, 97.6)	(6.51, 14.01)
Predicted risk ≥40%	75	12	86.2	95.7	86.2	95.7	19.90
	12	265	(77.1, 92.7)	(92.6, 97.7)	(77.1, 92.7)	(92.6, 97.7)	(11.37, 34.83)
Predicted risk ≥50%	71	4	81.6	98.6	94.7	94.5	56.51
	16	273	(71.9, 89.1)	(96.3, 99.6)	(86.9, 98.5)	(91.2, 96.8)	(21.25, 150.28)

Abbreviations: PPV, positive predictive value; NPV, negative predictive value; LR+, positive likelihood ratios; CI, confidence interval; IOTA SR, International Ovarian Tumor Analysis Simple Rules.

**Table 5:** Diagnostic indices of the combined IOTA Simple Rules models for postmenopausal women.

	Malignant	Benign	Sensitivity (%)	Specificity (%)	PPV (%)	NPV (%)	LR+
	*n*	*n*	(95%CI)	(95%CI)	(95%CI)	(95%CI)	(95%CI)
Postmenopausal women (*n* = 115)							
IOTA SR–refer women with inconclusive results							
Malignant and inconclusive	45	12	77.6	78.9	78.9	77.6	3.69
Benign	13	45	(64.7, 87.5)	(66.1, 88.6)	(66.1, 88.6)	(64.7, 87.5)	(2.19, 6.21)
IOTA SR X CA-125 for postmenopausal women							
Predicted risk ≥10%	57	9	98.3	84.2	86.4	98.0	6.22
	1	48	(90.8, 100.0)	(72.1, 92.5)	(75.7, 93.6)	(89.1, 99.9)	(3.41, 11.35)
Predicted risk ≥20%	56	8	96.6	86.0	87.5	96.1	6.88
	2	49	(88.1, 99.6)	(74.2, 93.7)	(76.8, 94.4)	(86.5, 99.5)	(3.61, 13.10)
Predicted risk ≥30%	55	7	94.8	87.7	88.7	94.3	7.72
	3	50	(85.6, 98.9)	(76.3, 94.9)	(78.1, 95.3)	(84.3, 98.8)	(3.85, 15.49)
Predicted risk ≥40%	55	6	94.8	89.5	90.2	94.4	9.01
	3	51	(85.6, 98.9)	(78.5, 96.0)	(79.8, 96.3)	(84.6, 98.8)	(4.22, 19.25)
Predicted risk ≥50%	55	5	94.8	91.2	91.7	94.5	10.81
	3	52	(85.6, 98.9)	(80.7, 97.1)	(81.6, 97.2)	(84.9, 98.9)	(4.67, 25.02)

**Abbreviations:** PPV, positive predictive value; NPV, negative predictive value; LR+, positive likelihood ratios; CI, confidence interval; IOTA SR, International Ovarian Tumor Analysis Simple Rules.

## Data Availability

The datasets used and/or analyzed during the current study are available from the corresponding author on reasonable request.

## Supplementary Materials

The following are available online at https://www.mdpi.com/2075-4418/11/2/173/s1, Table S1: Patient clinical characteristics and IOTA Simple Rules results in premenopausal women (*n* = 364), Table S2: Patient clinical characteristics and IOTA Simple Rules results in postmenopausal women (*n* = 115), Table S3: Histopathological classification of ovarian tumors in premenopausal and postmenopausal women, Table S4: Results of internal validation with bootstrap resampling procedures.
