# Supplementary material for: Diagnostic Added-Value of Serum CA-125 on the IOTA Simple Rules and Derivation of Practical Combined Prediction Models (IOTA SR X CA-125)
Source: Diagnostics (Basel). 2021 Jan 26;11(2):173. doi: 10.3390/diagnostics11020173 (PMC7912113; doi:10.3390/diagnostics11020173)
Supplement: Supplementary file 1 [file diagnostics-11-00173-s001.pdf]

Supplementary Table S1. Patient clinical characteristics and IOTA Simple Rules results in premenopausal women (n=364)

Parameters	Malignant (n=87)		Benign (n=277)		P-value
	n	(%)	n	(%)	
**Clinical characteristics**					
Age (year)*	36.3	±10.3	37.6	±9.0	0.260
Nulliparity	53	(60.9)	109	(39.4)	0.001
**Biomarker**					
Serum CA-125, (U/ml)**	384.9	(123.4, 708.2)	40.5	(24.0, 58.6)	<0.001
Range (min-max)		(16.4-1896.7)		(5.4-278.3)	
**IOTA Simple Rules features**					
Malignant tumor (M-features)					
M1	46	(52.9)	15	(5.4)	<0.001
M2	23	(26.4)	10	(3.6)	<0.001
M3	22	(25.3)	21	(7.6)	<0.001
M4	51	(58.6)	22	(7.9)	<0.001
M5	60	(69.0)	31	(11.2)	<0.001
Benign tumor (B-features)					
B1	5	(5.8)	145	(52.4)	<0.001
B2	0	(0)	13	(4.7)	0.044
B3	3	(3.5)	56	(20.2)	<0.001
B4	7	(8.1)	60	(21.7)	0.004
B5	24	(27.6)	244	(88.1)	<0.001
**IOTA Simple Rules diagnosis**					
Benign	6	(6.9)	208	(75.1)	NA
Malignant	59	(67.8)	19	(6.9)	NA
Inconclusive	22	(25.3)	50	(18.1)	NA

Abbreviations: IOTA, International Ovarian Tumor Analysis; SD, standard deviation; IQR, interquartile range; NA, not applicable.

*Mean±SD

**Median (IQR)

Supplementary Table S2. Patient clinical characteristics and IOTA Simple Rules results in postmenopausal women (n=115)

Parameters	Malignant (n=58)		Benign (n=57)		P-value
	n	(%)	n	(%)	
**Clinical characteristics**					
Age (year)*	59.0	±8.7	55.3	±7.8	0.018
Nulliparity	22	(37.9)	28	(49.1)	0.262
**Biomarker**					
Serum CA-125, (U/ml)**	492.8	(122.1, 732.1)	33.4	(20.4, 47.4)	<0.001
Range (min-max)		(13.7-2023.6)		(8.4-269.4)	
**IOTA Simple Rules features**					
Malignant tumor (M-features)					
M1	27	(46.6)	1	(1.8)	<0.001
M2	11	(19.0)	1	(1.8)	0.004
M3	10	(17.2)	5	(8.8)	0.268
M4	23	(39.7)	4	(7.0)	<0.001
M5	36	(62.1)	4	(7.0)	<0.001
Benign tumor (B-features)					
B1	4	(6.9)	24	(42.1)	<0.001
B2	1	(1.7)	4	(7.0)	0.206
B3	2	(3.5)	10	(17.5)	0.016
B4	10	(17.2)	18	(31.6)	0.085
B5	18	(31.0)	53	(93.0)	<0.001
**IOTA Simple Rules diagnosis**					
Benign	13	(22.4)	45	(79.0)	NA
Malignant	39	(67.2)	3	(5.3)	NA
Inconclusive	6	(10.3)	9	(15.8)	NA

Abbreviations: IOTA, International Ovarian Tumor Analysis; SD, standard deviation; IQR, interquartile range; NA, not applicable.

*Mean±SD

**Median (IQR)

Supplementary Table S3. Histopathological classification of ovarian tumors in premenopausal and postmenopausal women

Histopathological classification	Premenopausal (n=364)		Postmenopausal (n=115)		Overall (n=479)	
	n	(%)	n	(%)	n	(%)
**Malignant tumors**	**76**	**(20.8)**	**54**	**(46.9)**	**130**	**(27.2)**
Serous carcinoma	19	(5.2)	12	(10.4)	31	(6.5)
Endometrioid carcinoma	20	(5.5)	11	(9.6)	31	(6.5)
Mucinous carcinoma	2	(0.5)	15	(12.9)	17	(3.5)
Clear cell carcinoma	7	(1.9)	5	(4.4)	12	(2.5)
Yolk sac tumor	2	(0.5)	1	(0.9)	3	(0.6)
Dysgerminoma	4	(1.1)	0	(0)	4	(0.8)
Sex-cord stromal tumor	6	(1.7)	1	(0.9)	7	(1.5)
Other tumors	4	(1.1)	3	(2.6)	7	(1.5)
Metastatic carcinoma	12	(3.3)	6	(5.2)	18	(3.8)
**Borderline tumors**	**11**	**(3.1)**	**4**	**(3.5)**	**15**	**(3.0)**
Borderline serous tumor	5	(1.4)	1	(0.9)	6	(1.2)
Borderline mucinous tumor	6	(1.7)	3	(2.6)	9	(1.8)
Borderline endometrioid tumor	0	(0)	0	(0)	0	(0)
**Benign tumors**	**277**	**(76.1)**	**87**	**(49.6)**	**364**	**(69.8)**
Endometriotic cyst	95	(26.1)	11	(9.6)	106	(22.1)
Mature cystic teratoma	52	(14.3)	13	(11.3)	65	(13.6)
Mucinous cystadenoma	19	(5.2)	12	(10.4)	31	(6.5)
Serous cystadenoma	27	(7.4)	7	(6.1)	34	(7.1)
Pseudocyst	25	(6.9)	5	(4.4)	30	(6.3)
Leiomyoma	25	(6.9)	3	(2.6)	28	(5.8)
Other tumors	34	(9.3)	6	(5.2)	40	(8.4)

Supplementary Table S4. Results of internal validation with bootstrap resampling procedures

Parameters or statistics	Mean	SD	Min	Max
**Premenopausal model**				
Apparent AuROC	0.948113	0.017543	0.877739	0.991503
Apparent CITL	-0.00969	0.10411	-0.38092	0.276765
Apparent slope	8.354806	0.777098	6.58501	11.35269
Test AuROC	0.944209	0.0013	0.939541	0.947633
Test CITL	0.006266	0.101979	-0.28934	0.343617
Test slope	0.982131	0.066177	0.81173	1.220379
Optimism AuROC	0.003904	0.017454	-0.06898	0.046568
Optimism CITL	-0.01595	0.205918	-0.72454	0.566104
Optimism slope	0.017869	0.066177	-0.22038	0.18827
**Postmenopausal model**				
Apparent AuROC	0.977092	0.014082	0.919048	0.999455
Apparent CITL	0.009766	0.208748	-0.5625	0.566503
Apparent slope	9.995468	25.09239	5.428964	512.292
Test AuROC	0.976059	0.001861	0.968845	0.980944
Test CITL	-0.00614	0.213661	-0.52381	0.593935
Test slope	0.97512	0.087469	0.773721	1.262725
Optimism AuROC	0.001033	0.014131	-0.05645	0.024802
Optimism CITL	0.015903	0.421316	-1.15643	1.025843
Optimism slope	0.02488	0.087469	-0.26273	0.226279

Abbreviations: SD, standard deviation; Min, minimum; Max, maximum; AuROC, area under the receiver operating characteristic curve; CITL, calibration-in-the-large.
